# Feeding to satiation induces mild oxidative/carbonyl stress in the brain of young mice

**DOI:** 10.17179/excli2021-4347

**Published:** 2022-01-05

**Authors:** Oksana V. Kuzniak, Oksana M. Sorochynska, Maria M. Bayliak, Andrii Ya. Klonovskyi, Yulia V. Vasylyk, Halyna M. Semchyshyn, Kenneth B. Storey, Olga Garaschuk, Volodymyr I. Lushchak

**Affiliations:** 1 Department of Biochemistry and Biotechnology, Vasyl Stefanyk Precarpathian National University, 57 Shevchenko Str., Ivano-Frankivsk, 76018, Ukraine; 2 Institute of Biochemistry, Carleton University, 1125 Colonel By Drive, Ottawa, Ontario, K1S 5B6, Canada; 3 Department of Neurophysiology, Institute of Physiology, University of Tübingen, 72074 Tübingen, Germany

**Keywords:** defensive enzymes, carbonyl proteins, alpha-dicarbonyl compounds, every-other-day fasting, lipid peroxides

## Abstract

Intermittent fasting as a dietary intervention can prevent overweight and obesity in adult organisms. Nevertheless, information regarding consequences of intermittent fasting for redox status and reactive metabolite-mediated processes that are crucial for the normal functioning of organisms is limited. Since the information on effects of intermittent fasting on parameters of oxidative/carbonyl stress in the brains of young mice was absent, the present study addressed these questions using an every-other-day fasting (EODF) protocol. The levels of carbonyl proteins were ~28 %, 22 % and 18 % lower in the cerebral cortex of EODF males and females and middle parts of the brain of EODF males, respectively, as compared to their *ad libitum* fed counterparts. Lipid peroxides and α-dicarbonyl compounds were lower only in the cortex and medulla part of EODF male brain. The EODF regimen resulted in higher total non-specific antioxidant capacity in different parts of male brain and a tendency to be higher this parameter in females. At the same time, EODF regimen had no effect on the activities of the defensive antioxidant enzymes, namely superoxide dismutase, catalase, glutathione-S-transferase, glutathione peroxidase, glyoxylase 1 and glucose-6-phosphate dehydrogenase in the cortex of both sexes, but even decreased activities of these enzymes in medulla and middle part of the brain. In general, the results suggest that in the brain of young mice *ad libitum* feeding induces mild oxidative/carbonyl stress which may be partially alleviated by the EODF regimen. The effect of EODF regimen is more pronounced in the medulla part than in the cortex.

## Abbreviations

AL ad libitum

CP carbonyl proteins

EODF every-other-day fasting

G6PDH glucose-6-phosphate dehydrogenase

GPx glutathione peroxidase

GR glutathione reductase

GST glutathione-S-transferase

IDH isocitrate dehydrogenase

Kpi potassium phosphate buffer

LOOH lipid peroxides

RCS reactive carbonyl species

ROS reactive oxygen species

ROCS reactive oxygen/carbonyl species

TAC total antioxidant capacity

## Introduction

In the developed countries, most people consume food to satiation as do many laboratory and home animals. However, in evolutionary terms, this is not a normal life style because unlimited access to food is relatively rare in nature and such times are usually interrupted by varying periods of starvation when food is scarce. Through natural selection, animals have evolved mechanisms to efficiently accumulate resources in order to survive periods of starvation. In developed human societies or with laboratory, pet or many farmed animals excessive food intaken is accumulated as reserves due to the evolutionary developed strategy. In most cases, excess food consumption leads to fat storage (Sandouk and Lansang, 2017[37]; Bayliak et al., 2019[4]). 

Overweight is known to be associated with increased generation of reactive oxygen species (ROS) and reactive carbonyl species (RCS) (which collectively will be abbreviated here as ROCS) leading to the development of oxidative/carbonyl stress (Uribarri, 2017[48]; Anderson et al., 2018[2]; Bayliak et al., 2019[4]). This causes increased production of pro-inflammatory cytokines (Muñoz and Costa, 2013[30]). Oxidative/carbonyl stress may be defined as a transient or persistent increase in steady-state ROCS levels, disturbing cellular core and signaling pathways, including ROCS-based ones, and leading to chemical modification of cellular constituents which, if not counterbalanced, may culminate in cell death via necrosis or apoptosis. Reactive species can interact with virtually any components of the organism. Under normal conditions ROCS generation is counterbalanced by an adequate defensive antioxidant/antiglycation system. However, oxidative/carbonyl stress can be associated with a number of pathologies, although it is not usually clear if they are the cause or the consequence of the stress.

**Figure 1:**
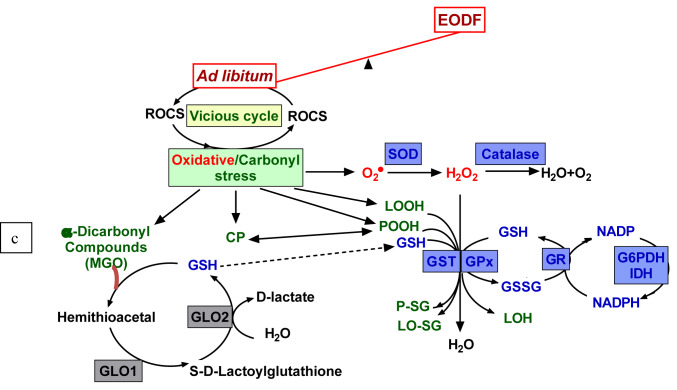
Relationship between feeding regimen, ROCS production, and protective mechanisms. Reactive oxygen (ROS) and carbonyl species (RCS) are constantly produced as byproducts of non-enzymatic oxidation/glycation and can interact with virtually all cellular components leading to development of oxidative/carbonyl stress. Lipid peroxides (LOOH), protein peroxides (POOH) and α-dicarbonyl compounds (MGO - methylglyoxal) are reactive intermediate products and carbonyl proteins (CP) are the end products of glycoxidation, and, therefore, they are the hallmarks of oxidative/carbonyl stress. Under normal conditions, a balance between ROCS generation and elimination is maintained by complex antioxidant/antiglycation defensive systems. Feeding to satiation leads to an increase in ROCS production followed by chronic oxidative/carbonyl stress with intensification of the modification of biomolecules. Every-other-day fasting (EODF) regimen alleviates the intensity of oxidative/carbonyl stress preventing biomolecule damages. Denotations of defensive enzymes: GLO1 and GLO2 - glyoxalase 1 and 2, SOD - superoxide dismutase, GST - glutathione-S-transferase, GR - glutathione reductase, GPx - glutathione peroxidase. G6PDH - glucose-6-phosphate dehydrogenase, IDH - isocitrate dehydrogenase.

Recent studies have linked oxidative/carbonyl stress to detrimental effects of overeating and overweight-related pathologies (Uribarri, 2017[48]; Anderson et al., 2018[2]; Petriv et al., 2021[34]). Non-enzymatic oxidation/glycation (glycoxidation) and reactive oxidative/carbonyl species (ROCS) are believed to underlie the stress-mediated modification of biomolecules and nutrients, particularly proteins, lipids and carbohydrates (Figure 1(Fig. 1)). Lipid peroxides (LOOH) and α-dicarbonyl compounds are reactive intermediate products and protein carbonyls are end products of glycoxidation, and, therefore, they are hallmarks of oxidative/carbonyl stress. These glycoxidation products have been found to generate ROCS (Ott et al., 2014[33]), which, in turn, start new rounds of non-enzymatic interactions and modifications. This suggests that the antioxidant/antiglycative system might inhibit the development of the vicious cycle (Figure 1(Fig. 1)). Here we examine the potential beneficial effects of intermittent fasting to prevent a detrimental cascade of non-enzymatic reactions and to induce mild oxidative/carbonyl stress stimulating defensive mechanisms to break down the detrimental vicious cycle.

Limited access to food and moderate physical activity are virtually the only reliable non-pharmacological strategies known to date to fight overweight (Lushchak and Gospodaryov, 2018[19]). Prophylactic and curative effects of controlled starvation have been known since ancient times and are an organic component of virtually all religions (Koufakis et al., 2017[15]; Venegas-Borsellino et al., 2018[50]). There are different regimens of controlled starvation but in all cases the absence of malnutrition is important. For humans, intermittent fasting is probably the most physiological and psychologically acceptable approach to promote dietary calorie restriction (Mattson, 2019[27]). 

The present study aimed to fill a gap in our knowledge about the response of young animals to intermittent fasting by investigating effects of one-month of an every-other-day fasting (EODF) regimen on one-month-old mice with a focus on processes involving reactive oxygen/carbonyl species. Moreover, since most available publications used male mice, we decided to test both sexes in order to decipher sex specificity in the response to the EODF treatment.

## Materials and Methods

### Reagents

Most reagents including 2,2'-azinobis(3-ethylbenzothiazoline-6-sulfonic acid) diammonium salt (ABTS), bovine serum albumin, 1-chloro-2,4-dinitrobenzene, cumene hydroperoxide, 2,4-dinitrophenylhydrazine (DNPH), 5,5′-dithiobis-2-nitrobenzoic acid, ferrous sulfate, glucose-6-phosphate, L-glutathione reduced, L-glutathione oxidized, glutathione reductase, DL-isocitric acid trisodium salt hydrate, magnesium chloride hexahydrate (MgCl_2 _× 6 H_2_O), methylglyoxal solution, potassium dihydrogen phosphate (KH_2_PO_4_), S-lactoylglutathione, sodium chloride (NaCl), N,N,N′,N′-tetramethylethylenediamine (TEMED), quercetin, 6-hydroxy-2,5,7,8-tetramethychroman-2-carboxylic acid (Trolox), and xylenol orange were purchased from Sigma-Aldrich (USA). Ethylenediamine-tetraacetic acid (EDTA), 2-(N-morpholino)ethanesulfonic acid (MES) hydrate, NADP^+^, NADPH, phenylmethylsulfonyl fluoride, and Tris were from Carl Roth (Karlsruhe, Germany). All other reagents were obtained from local suppliers (Ukraine) and were of analytical grade.

### Animals and experimental conditions 

The mice used in this study were C57BL/J6 strain kindly provided by Dr. I. Shmarakov from Yuriy Fedkovych Chernivtsi National University (Chernivtsi, Ukraine) and were then bred in our department facilities. All animals were bred in a closed colony housed under standard vivarium conditions (12-h light/dark cycle, 22±2 °C temperature, and 50-60 % humidity). 

For experiments, one-month-old mice were separated by sex and randomly assigned to control or experimental groups of 5-6 mice per cage. Mice in the control group had *ad libitum *(AL) access to food, whereas mice in experimental groups were subjected to an intermittent fasting regimen (EODF) that consisted of free access to food for 24 h followed by food deprivation for the next 24 h. Food was provided to the EODF groups at 9 a.m. and withdrawn at 9 a.m. the next morning. All mice received regular chow containing 21.8 % protein, 4.8 % fat, 69.1 % carbohydrates, and 3.9 % fiber (“Rezon-1”, Kyiv, Ukraine) and had unlimited access to water. Animals were kept on their feeding regimen under standard vivarium conditions for one month. All experimental protocols were approved by the Animal Experimental Committee of Vasyl Stefanyk Precarpathian National University. 

### Tissue collection and extraction procedure

After one month on either control or experimental feeding protocols, food was withdrawn for at 9 a.m. prior to euthanasia between 11-12 a.m. Experimental mice were sampled on the morning after their last day of fasting. Animals were euthanized using light carbon dioxide gas anesthesia. The brain was rapidly dissected and split into three parts, namely the cerebral cortex, middle part, and medulla part. The middle part included midbrain, substantia nigra, hippocampus, thalamus, hypothalamus, caudate putamen, basal forebrain, ventral striatum, and anterior olfactory nucleus. The medulla part of the brain included cerebellum, pons, and medulla oblongata. All brain parts were quickly rinsed in 0.9 % cooled NaCl solution and then frozen quickly in liquid nitrogen (-196 °C) followed by transfer to -80 °C for storage. All analyses used frozen tissue samples.

Frozen tissues were weighed and homogenized at 1:10 (w:v) ratio in cold homogenization medium containing 50 mM potassium phosphate buffer (KPi, pH 7.5), 0.5 mM EDTA, and 1 mM phenylmethylsulfonyl fluoride. Homogenates were then centrifuged (16,100 *g*, 15 min, 4 °C) in an Eppendorf 5415 R centrifuge (Hamburg, Germany) and supernatants were used for all biochemical assays, except lipid peroxide evaluation.

### Assays of carbonyl proteins, α-dicarbonyl compounds, and lipid peroxides

Levels of carbonyl groups in proteins (CP) were measured by reaction of carbonyl groups with 2,4-dinitrophenylhydrazine (DNPH) leading to formation of dinitrophenylhydrazones with an absorbance peak at 370 nm (Levine et al., 2000[16]; Lushchak et al., 2012[26]). Proteins in supernatants were precipitated with 30 % trichloroacetic acid and then treated with 10 mM DNPH as described earlier (Lushchak et al., 2012[26]). Results were expressed as nanomoles of carbonyl groups per milligram of protein.

Levels of lipid peroxides (LOOH) were measured by the FOX (ferrous-xylenol orange) method using cumene hydroperoxide as a positive standard (Lushchak et al., 2012[26]). Lipid peroxides were extracted by tissue homogenization in cold 96 % ethanol in a ratio 1:5 (w:v). After centrifugation (10,000 *g*, 10 min, 4 °C), the supernatants were used for LOOH assay as previously described (Lushchak et al., 2012[26]). Levels of LOOH were expressed as nanomoles of cumene hydroperoxide equivalents per gram of wet mass.

Levels of α-dicarbonyl compounds were determined in supernatants by their interaction with the Girard-T reagent in 30 mM sodium tetraborate buffer (pH 9.2). The optical density of the complex formed was determined at 325 nm using an extinction coefficient of 18.8 mM^−1^ cm^−1^ for Girard's hydrazone derivative of glyoxal (Mitchel and Birnboim, 1977[29]; Semchyshyn et al., 2014[40]). The results were expressed in nanomoles of glyoxal equivalents per milligram of protein. 

### Assay of total antioxidant capacity 

Total antioxidant capacity (TAC) of supernatants was measured by scavenging ABTS^• +^ cation radical (Erel, 2004[8]; Vasylkovska et al., 2015[49]). ABTS^• +^ cation radical resulted from the interaction of 2,2'-azino-bis (3-ethylenbenzozylin-6-sulfonate) (ABTS) with H_2_O_2_ in acetate buffer (pH 3.6). The disappearance of ABTS^• +^ was monitored at 414 nm using a Multiskan MCC/340 microplate reader (Labsystems, Helsinki, Finland). Solutions of Trolox (ranging from 9 to 90 µM) were used as standards for the calibration curve. The results were expressed as nanomoles of Trolox equivalents per milligram of protein.

### Measurement of activities of antioxidant and associated enzymes 

Activities of superoxide dismutase (SOD), catalase, glutathione reductase (GR), glutathione peroxidase (GPx), glutathione-S-transferase (GST), glucose-6-phosphate dehydrogenase (G6PDH), and NADP-dependent isocitrate dehydrogenase (IDH) were measured as described previously (Lushchak et al., 2005[22], 2008[21]). The activity of glyoxalase I (GLO1) was measured by monitoring the formation of S-D-lactoylglutathione at 240 nm (Ratliff et al., 1996[35]; Semchyshyn et al., 2014[40]). The reaction mixture for GLO1 activity measurement contained 100 mM MES [2-(N-morpholino)ethanesulfonic acid] buffer (pH 6.5), 1 mM GSH, 8 mM methylglyoxal, and 20 μl supernatant in a final volume of 1.5 ml. Aconitase activity was measured by the formation of cis-aconitate acid from isocitrate at 240 nm (Hausladen and Fridovich, 1994[12]). The reaction mixture contained 50 mM KPi buffer (pH 7.5), 1 mM isocitrate, 0.6 mM MnCl_2_, and 30 μl of supernatant in a final volume of 1.6 ml. 

The activities of catalase, aconitase and GST were calculated using the extinction coefficients for H_2_O_2_ (39.4 M^-1^ cm^-1^), cis-aconitate (3.6 mM^-1^ cm^-1^), and an adduct of GSH and 1-chloro-2,4-dinitrobenzene (9.6 mМ^-1^см^-1^), respectively. The extinction coefficient for NADPH of 6.22 mM^-1^ cm^-1^ was used for calculations of GPx, GR, G6PDH, and IDH activities. The extinction coefficient for S-lactoylglutathione of 3.1 mM^-1^ cm^-1^ was used for calculations of GLO1 activity. One unit of SOD activity was defined as the amount of enzyme (per milligram of protein) that inhibited quercetin oxidation by 50 % of the maximal inhibition. Inhibition values for SOD activity were calculated using an Enzyme Kinetics computer program (version 3.1) (Brooks, 1992[6]). One unit of other enzyme activities was defined as the amount of the enzyme consuming 1 μmol of substrate or generating 1 μmol of product per minute; the activities were expressed as international units per milligram of protein (U/mg protein). Soluble protein concentrations were determined by the Bradford assay (Bradford, 1976[5]) using bovine serum albumin as the standard. 

### Statistical analysis 

Data are presented as means ± SEM. Statistical analysis was performed using a two-tailed Student's t-test using the Mynova computer program (version 1.3) (Brooks, 1992[6]). 

## Results

Over 90 % of rodent studies analyzing effects of dietary restriction on oxidative stress markers were carried out on male animals only (Walsh et al., 2014[51]). In the present work we used mice of both sexes in order to evaluate any gender specificity of EODF bioeffects. We used young mice (4 weeks old) that were subjected for four weeks to regimens of either *ad libitum* (AL) feeding (control) or intermittent fasting applied as EODF (experimental). Animal body mass characteristics and data about food consumption during the experiment were provided in our previous study (Sorochynska et al., 2019[45]). Briefly, at the final stage on the experiment on average, AL males consumed 27 % more calories than their EODF counterparts (12.2 kcal/day AL and vs 8.9 kcal/day EODF), whereas AL females consumed 25 % more calories than their EODF counterparts (10.5 kcal/day AL and vs 7.8 kcal/day EODF) which well corresponds to literature data for AL groups (List et al., 2013[17]; Yang et al., 2014[53]). EODF mice were significantly lighter than controls by 14 and 13 % for males and females, respectively (Sorochynska et al., 2019[45]; Supplementary Figure 1). These data collectively clearly testify that EODF animals of both sexes were dietary restricted to virtually the same extent. 

Here, we aimed to compare free radical and glycoxidation processes in the brains of the two mouse groups. To do so, we characterized several parameters characterizing oxidative/carbonyl stress indices processes in three parts of the brain: the cerebral cortex, middle part (containing midbrain, substantia nigra, hippocampus, thalamus, hypothalamus, caudate putamen, basal forebrain, ventral striatum and anterior olfactory nucleus), and medulla part (medulla oblongata, cerebellum, and pons). 

### Intermittent fasting lowered intensity of oxidative/carbonyl stress 

**Figure 2:**
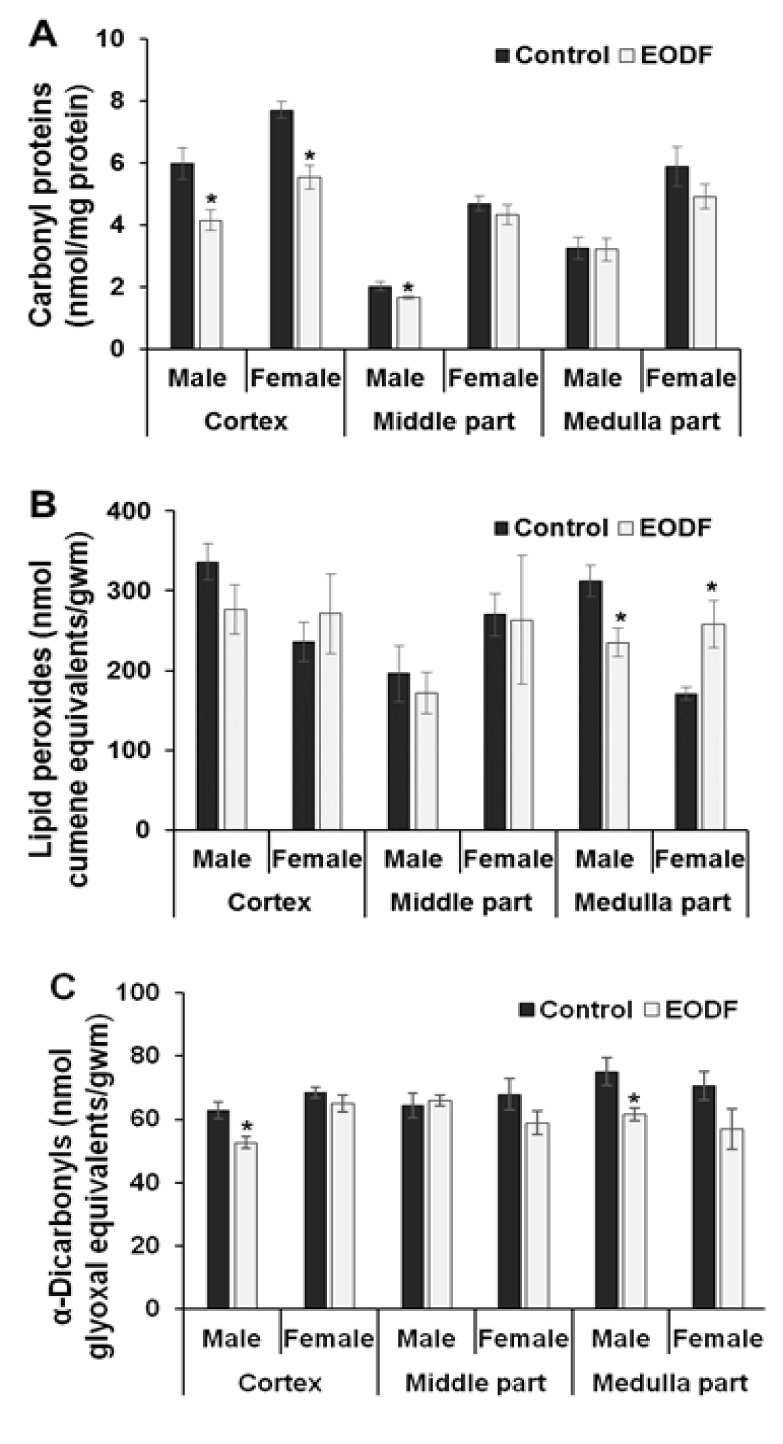
The effect of EODF on the levels of markers of oxidative/ carbonyl stresses in different parts of the brains of young mice: (A) carbonyl groups in proteins (*n* = 4-6), (B) lipid peroxides (*n* = 3-6), and (C) α-dicarbonyl compounds (*n* = 5-6). Data are mean ± SEM. *Significantly different from the corresponding values for the *ad libitum* fed (control) group, *P *< 0.05.

Measurement of the levels of oxidative/carbonyl stress markers such as carbonyl proteins (CP), lipid peroxides (LOOH), and α-dicarbonyl compounds is probably the most common approach to indirectly evaluate the intensity of ROCS-related processes (Lushchak et al., 2012[26]; Semchyshyn et al., 2014[40]; Sies et al., 2017[44]; Garaschuk et al., 2018[11]). Figure 2(Fig. 2) shows the levels of these markers in the brains of control (AL) and experimental (EODF) animals, both males and females. Among all three brain parts studied, the cortexes of EODF mice of both sexes and the middle brain parts of EODF males possessed lower CP levels (reduction by ~30 %, 28 %, and 18 %, respectively) compared to AL animals. In addition, EODF treatment resulted in a tendency to decrease CP levels in the female middle and medulla parts of the brain, and did not affect CP content in the medulla parts of experimental males (Figure 2A(Fig. 2)). The levels of LOOH tended to be lower in the cortexes and middle parts or significantly decreased (33 %) in the medullas of EODF males. In EODF females LOOH levels were even higher (51 %) in the brain medullas, demonstrated a tendency to increase in the cortexes, and were not affected in the middle parts of the brains as compared to AL controls (Figure 2B(Fig. 2)). The levels of α-dicarbonyl compounds were 16 % and 18 % lower in the cortexes and medullas of experimental males compared with respective AL values. EODF females showed a tendency for α-dicarbonyl compounds to decrease in the middle and medulla parts of the brain compared with AL values (Figure 2C(Fig. 2)). 

The enzyme aconitase that possesses an [Fe-S]-cluster in its active center is sensitive to oxidation by ROS and carbonylation by RCS. The latter cause the reduction of enzymatic activity (Liu et al., 2013[18]; Lushchak et al., 2014[20]). Therefore, in this study we measured aconitase activities in mouse brains as a marker of oxidative/carbonyl stress and found that they were lower in the middle part of female brains or tended to be lower in female cortexes and male middle and medulla parts under the EODF regimen (Table 1(Tab. 1)). EODF regimen provided 29 % lower aconitase activity than in control only in middle parts of females (Table 1(Tab. 1)). 

Generally, under most conditions studied, the parameters of oxidative/carbonyl stress were significantly lower or tended to be lower in the brains of the EODF experimental animals.

### Intermittent fasting slightly increased total antioxidant potential

In all parts of the brain exposured to the EODF regimen resulted in higher total antioxidant capacity compared to AL controls but the differences were significant only in the male cortexes and medulla parts, where the values for EODF were 44 % and 33 % higher than in AL, respectively (Table 2(Tab. 2)).

### The activities of defensive enzymes were either lowered or not changed in fasting mice

The above data on the parameters of oxidative/carbonyl stress, aconitase activities and total antioxidant capacity suggest that intermittent fasting lowers the intensity of these stresses in the brains of the experimental mice. This prompted us to search for changes in the activities of defensive enzymes that could be responsible for this effect (Figure 1(Fig. 1)). We analyzed activities of the following enzymes: (*i*) antioxidant - superoxide dismutase (SOD), catalase, and glutathione peroxidase (GPx), and (*ii*) antiglycation - glyoxalase 1 (GLO1). 

**Figure 3:**
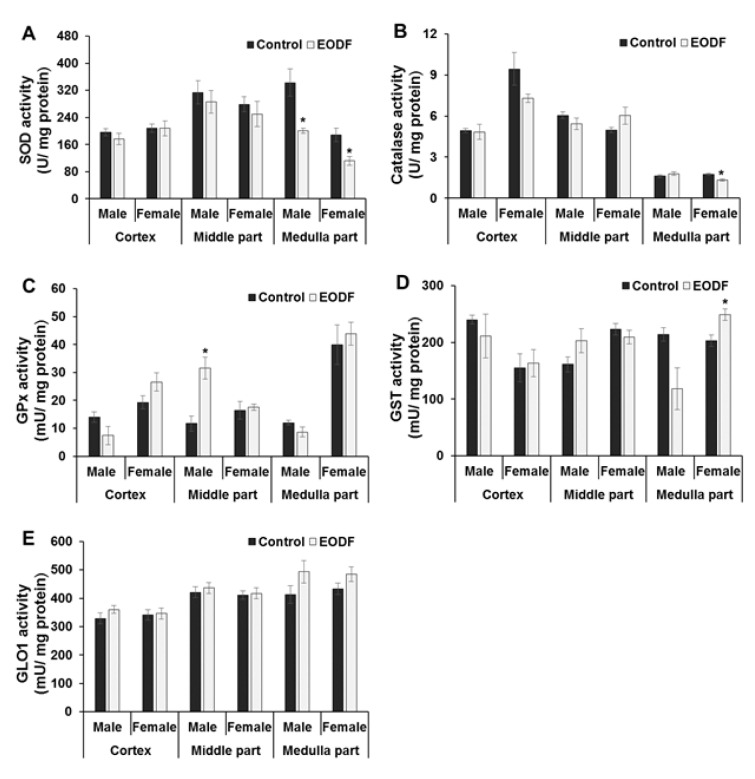
The effect of EODF on the activities of: (A) superoxide dismutase (*n* = 3-6), (B) catalase (*n* = 5-6), (C) glutathione peroxidase (*n* = 4-6), (D) glutathione-S-transferase (*n* = 4-6), and (E) glyoxalase 1 (*n* = 5-6) in different parts of the brain of male and female young mice. Data are mean ± SEM. *Significantly different from the corresponding values for the *ad libitum* fed (control) group, *P *< 0.05.

The activities of primary antioxidant enzymes SOD and catalase did not differ substantially between AL and EODF groups. For example, the activities of SOD were lower by 44 % in medulla parts of the brain of EODF animals compared with AL controls and also tended to be lower in the middle parts but were not affected in the cortex of EODF animals of either sex as compared to respective controls (Figure 3A(Fig. 3)). Enzymatic activities of catalase were virtually unaffected by EODF and only in female medullas were activities significantly (by 25 %) lower than in respective AL groups (Figure 3B(Fig. 3)). 

Like SOD and catalase, GPx belongs to the first-line detoxifying ROS defenses because it can directly convert hydrogen peroxide to water (Figure 1(Fig. 1)). At the same time, GPx is a secondary antioxidant enzyme because it detoxifies secondary products of ROS modification; for instance, using GSH as a co-substrate, GPx reduces lipid peroxides (LOOH) to respective alcohols (LOH). The activities of GPx were not generally affected by the EODF regimen (Figure 3C(Fig. 3)), only male middle brain parts of EODF animals showed significantly (2.7-fold) higher GPx activity than those in the AL control animals. In other cases, the activities tended to be lower in the cortexes and medullas of EODF males and did not differ significantly between control and EODF females (Figure 3C(Fig. 3)). 

Glutathione-S-transferase (GST), like GPx, needs GSH as a co-substrate and belongs to the secondary antioxidant defense system, reducing organic LOOH, but not hydrogen peroxide (Figure 1(Fig. 1)). The activities of GST did not change significantly under EODF treatment in the cortexes or middle brain parts, whereas in the medullas GST concentration tended to be lower (by 45 %) in males but significantly higher (by 23 %) in EODF females compared to those in control groups (Figure 3D(Fig. 3)). Glyoxalase 1 (GLO1) is a key antiglycation GSH-dependent enzyme, which is responsible for detoxification of alpha-dicarbonyl compounds (Figure 1(Fig. 1)). Analysis of GLO1 activities found no significant differences between AL and EODF mouse groups in any of the three brain parts tested (Figure 3E(Fig. 3)). 

### The activities of enzymes associated with antioxidant and antiglycation systems were little changed by intermittent fasting

Selected defensive enzymes depend on the reducing power provided by glutathione (GSH), which is generated by glutathione reductase (GR). In turn, regeneration of oxidized glutathione (GSSG) to GSH relies on NADPH produced by glucose-6-phosphate dehydrogenase (G6PDH) and NADP-dependent isocitrate dehydrogenase (IDH) (Lushchak, 2012[26]) (Figure 1(Fig. 1)). In concert, these enzymes maintain the glutathione pool in a reduced state. 

**Figure 4:**
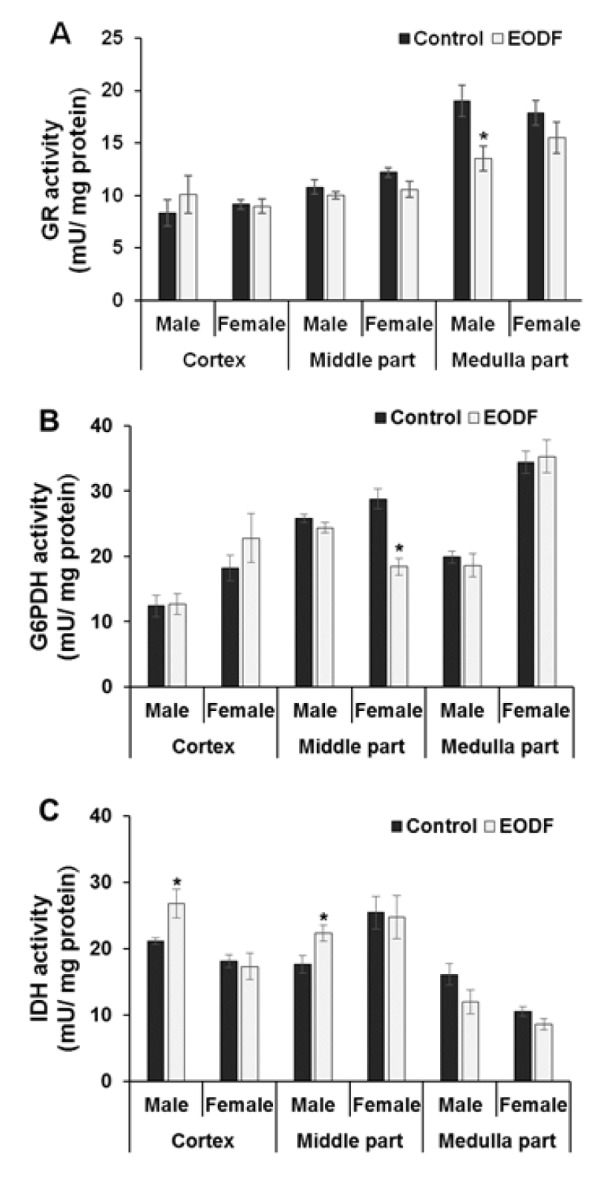
The effect of EODF on the activities of the enzymes responsible for maintaining glutathione redox status: (A) glutathione reductase (*n* = 5-6), (B) glucose-6-phosphate dehydrogenase (*n* = 4-6), and (C) NADP-dependent isocitrate dehydrogenase (*n* = 4-6) in different parts of the brain of young mice. Data are mean ± SEM. *Significantly different from the corresponding values for *ad libitum* fed (control) group, *P *< 0.05.

The activities of three enzymes involved in maintaining of glutathione pool in reduced state were also assessed in the brains of AL versus EODF mice. Exposure to EODF resulted in 29 % lower GR activities in the medulla parts of male mice than in AL animals but no significant differences were found in the cortex or middle parts of male brains. The female mice showed no differences in GR activities (Figure 4A(Fig. 4)). 

The activities of G6PDH were 36 % lower in the brain middle parts of EODF females compared to their AL counterparts but no other significant differences between control and experimental groups were found (Figure 4B(Fig. 4)). Exposure to EODF resulted in 27 % higher IDH activities in the cortex and middle parts of the male brains than in AL ones but no other differences were found (Figure 4C(Fig. 4)). 

## Discussion

This work extends the knowledge on the effects of dietary restriction caused by an every-other-day fasting regime (EODF) to young animals, both males and females. To our best knowledge, such data are absent in the literature. Here we used not commonly applied design because we housed animals not individually in the cages but in groups for 5-6 in the cage. The advantage of such EODF protocol includes: (*i*) the ability to keep animals within their social group in order to avoid stress which animals are subjected at individual housing and (*ii*) free and *ad libitum* access to food on the feeding day, so that each mouse may consume forage without competing or fighting. Earlier we reported that at this design experimental mice of both sexes were dietary restricted (Sorochynska et al., 2019[45]).

For a long time, it has been known that dietary restriction perturbs virtually all functions of living organisms changing the homeostasis to a more economic use of resources. Because energy is needed to power virtually all body functions and dietary restriction influences these functions, attention was first paid to the question: “How animals change their metabolism to meet energy demands”? The question is generally answered with a description of cooperation between organs to redirect resources to maintaining the operation of the most crucial organs needed for survival - the brain (Rothman and Mattson, 2013[36]; Amigo and Kowaltowski, 2014[1]) and the heart (Seals et al., 2018[38]). Operation of a variety of other functions, such as growth and reproduction, may be temporally suppressed. 

The majority of rodent and human studies have generally demonstrated that dietary restriction (intermittent and short-term fasting) decreased plasma glucose and other parameters of carbohydrate metabolism, whereas fat oxidation was increased (Tinsley and La Bounty, 2015[47]; Antoni et al., 2017[3]; Sorochynska et al., 2019[45][46]; Freire et al., 2020[10]). Lipid oxidation, in turn, significantly stimulates ROCS formation, resulting in the development of carbonyl/oxidative stress (Niki, 2009[31]; Onyango, 2012[32]; Semchyshyn, 2014[39]; Garaschuk et al., 2018[11]). 

Intermittent fasting was found to enhance oxygen consumption by mice and release of CO_2_ and heat (Xie et al., 2017[52]). This clearly demonstrates an intensification of aerobic metabolism. Since over 90 % of oxygen consumed by animals is used by the mitochondria for ATP production (Jády et al., 2016[13]), there are no doubts that excessive oxygen consumed during EODF is mostly used by mitochondria. Augmentation of oxygen consumption by mitochondria may result in increased ROS generation as side products of operation of the mitochondrial electron transport chain, as mitochondria produce over 90 % of the cell's ROS (Cadenas and Davies, 2000[7]). Although spatiotemporal distribution of sites of ROS production and elimination cannot be ignored, a steady-state ROS level is usually used to describe the ROS homeostasis (Lushchak, 2015[24]; Siegrist and Sies, 2017[41]; Sies, 2017[44]). 

It is logical to suggest that perturbation of metabolism by EODF may result in enhanced ROCS generation and cause oxidative/carbonyl stress. Most investigations on the relationship between dietary restriction and oxidative stress in rodents were discussed in an excellent review by Walsh and colleagues (2014[51]). Therefore, we will rely on the virtually encyclopedic information from the paper in this discussion and integrate our results into this framework.

Cells strive to keep the level of reactive species under strict control. Generation and elimination of ROCS are usually well balanced and perturbations of the balance may cause redox stress. An increase in the steady-state ROS level results in oxidative stress, whereas a decrease in the ROS level can lead to reductive stress (Lushchak, 2015[24]; Sies, 2015[43]; Sies et al., 2017[44]). Like ROS, RCS play a dual role in living systems and modulate different biological processes, therefore RCS levels are tightly controlled *in vivo* (Semchyshyn, 2014[39]). Thus, it seems that the balance between the production and elimination of ROS is more critical than their absolute values.

It should be underlined again that this work was designed to evaluate effects of dietary restriction caused EODF in young mice and compared with effects described for adult animals. Levels of markers of oxidative/carbonyl stress, namely CP, LOOH and α-dicarbonyl compounds are the most broadly used markers for routine evaluation of the intensity of oxidative/carbonyl stress. In this study, these parameters showed significant reductions or downward trends in EODF compared with AL mice in almost all instances (Figures 2A, B, and C(Fig. 2)). This corresponds well with most rodent and human studies on the effects of dietary restriction (Zakaria et al., 2013[54]; Walsh et al., 2014[51]). At the same time, the aconitase activities tended to be lower (Table 1(Tab. 1)) and total antioxidant capacities tended to be higher (Table 2(Tab. 2)) in the brains of EODF compared with AL mice. These data clearly show that the *brains of EODF animals were subjected to less intensive oxidative and carbonyl stresses than those of their control AL counterparts*. 

Further support for the above statement came from measurement of activities of primary antioxidant enzymes, namely SOD and catalase. In some cases (e.g., SOD in both male and female medulla; catalase in female medulla), the activities of these enzymes in the brains of EODF mice were lower than those in AL controls but in no cases were these activities significantly higher in EODF brain (Figures 3A, B(Fig. 3)). The activities of GPx and GST varied between EODF and AL groups but no consistent pattern emerged, whereas GLO1 activities remained constant in all cases (Figures 3C, D, and E(Fig. 3)). It should be noted that the activities of key antioxidant enzymes in mouse brain are increased between birth and maturation (Mavelli et al., 1982[28]; Khan and Black, 2003[14]). 

The lower or unchanged activities of the principal antioxidant enzymes, SOD and catalase, in EODF animals contradict most other studies carried out to date (Walsh et al., 2014[51]). These data, along with our data on the levels of CP, LOOH and alpha-dicarbonyls and aconitase activities, suggest that the intensity of oxidative/carbonyl stress in the brains of EODF animals was lower than that in AL animals. The other parameters associated with antioxidant/antiglycation enzymes defensive against oxidative/carbonyl stress, namely GR, G6PDH, and IDH activities, varied slightly between experimental and control groups, but no strict tendency to change could be discerned from analysis of the data. 

The literature data, showing increases or no changes in activities of defensive enzymes, look reasonable because it is known that EODF enhances oxygen consumption (Xie et al., 2017[52]). Therefore, one could expect more extensive ROS production in young EODF animals. However, unexpectedly, lower ROS production by mitochondria isolated from fasting animals was found in 60 % of studies and was unchanged in 40 % (Walsh et al., 2014[51]). Caloric restriction usually results in lower carbohydrate reserves (glycogen and glucose) but triacylglyceride levels were slightly higher in liver and unchanged in the brain cortexes of EODF mice relative to AL fed mice (Sorochynska et al., 2019[45]). Moreover, concentrations of ketone bodies were also lower in the liver, but not in the brain of EODF mice of the same strain (Sorochynska et al., 2019[45]). This may suggest enhanced RCS generation and carbonyl stress development in fasting animals. Clearly, fasting animals are more physically active than AL ones as evidenced by their higher oxygen consumption (Xie et al., 2017[52]). Because ROS production by mitochondria is measured *in vitro* with isolated preparations and is normalized to protein levels (Federico et al., 2012[9]), enhanced ROS generation due to higher oxygen consumption may not take place *in situ*. Unchanged or enhanced activities of defensive antioxidant/antiglycation and associated enzymes may indicate effects of ROCS-mediated modifications of biomolecules that fit well with the accepted point of view on balanced and coordinated processes of ROCS generation and elimination leading to probable steady-state ROCS levels (Lushchak, 2015[24]; Sies, 2015[43]; Garaschuk et al., 2018[11]).

Confirming most studies that show fasting-promoted decreases in the content of oxidatively modified proteins and lipids, we also found lower activities of principal defensive enzymes. Because sex effects were minor (i.e. in most cases animal responses to EODF were similar in males and females), our further discussion will concentrate more on the mouse strain and age. Most previous studies were carried out using mature male adult C57BL/6 strain mice or other strains, whereas we used a mixed C57BL×sv129 strain. In this work we did not measure oxygen consumption in mice but we confirmed most literature data on levels of energy substrates and the operation of mitochondria (Sorochynska et al., 2019[45]). This shows that our strain and experimental design are generally similar to those described so far (Xie et al., 2017[52]). Collectively, our data may show that the brains of young mice (of the strain used) exposed to EODF experienced lower intensity of oxidative stress relative to those on the AL regimen. This conclusion is supported not only by lower levels of ROS-modified molecules but also by lower activities of antioxidant enzymes. We suggest that in our case young EODF animals were able to reduce ROS-promoted damage more efficiently than older animals due to which there was no need to markedly enhance activities of antioxidant enzymes.

**Figure 5:**
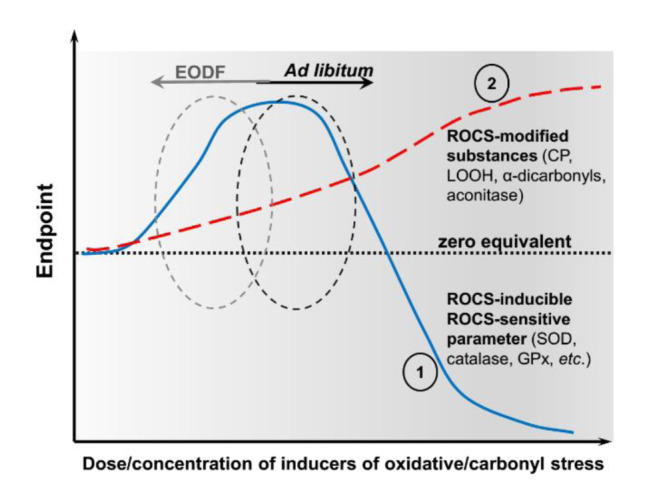
Schematic presentation of the results showing Endpoint responses of parameters of interest (ordinate) to varied dose/concentration of inducers of oxidative stress (abscissa). Line 1 (blue colored) shows dependence on the levels of ROCS-modified cellular component/s such as CP, LOOH, α-dicarbonyl compounds, and aconitase in this study, and line 2 (red colored) shows dependence on the levels of ROCS-activated and simultaneously ROCS-sensitive components such as the activities of superoxide dismutase, catalase, and GPx in this study. Black and grey circles on curves 1 and 2 correspond to *ad libitum* and EODF fed animals, respectively.

Our present data and that reviewed by Walsh and colleagues (2014[51]) can be explained from the point of view of the proposed recently intensity-based classifications of oxidative/carbonyl stress (Lushchak, 2015[24]; Garachchuk et al., 2018[11]). This is illustrated schematically in Figure 5(Fig. 5). There are two curves in this graph: 1 - ROCS-modified cellular components (in our work, these are represented by CP, LOOH, α-dicarbonyls and aconitase), and 2 - ROCS-activated and ROCS-sensitive components represented here, for example, by the activities of superoxide dismutase and catalase. In our experiments, the brains of AL fed animals showed higher levels of ROCS-modified components than mice in the EODF experimental groups due to the shift to the right of curve 1 relative to the EODF 6 data located to the left of AL. There may be at least two reasons why such situation occurs. First, we used very young animals (one month old at the beginning of the experiment), which hints at age-specific differences, and second, we used a mouse strain that has not previously been studied from this point of view. 

Two more conclusions may be drawn from our work: (1) all three parts of the mouse brains responded to the EODF regimen very similarly, and (2) both sexes showed rather similar responses under the EODF regimen, with no substantial differences between males and females. In order to clarify if there is some strain-specific mouse response to EODF we suggest to carry out further similar studies with young mice of other strain/s, for example, with the C57BL/6 parental strain to that used in the current study.

## Conclusions and Perspectives

It is generally accepted that animals fed an AL diet are appropriate controls for most experimental situations including studies of dietary calorie restriction. Even normally fed animals slowly increase their mass during life, similar to humans. Frequently such animals can become overweight. In humans, this causes or worsens many age-related pathologies like cardiovascular diseases, cancer, diabetes, neurodegeneration, *etc*. Such conditions are known to be accompanied by oxidative/carbonyl stress, which enhances cell damage creating a vicious cycle resulting in enhanced tissue injuries (Figure 1(Fig. 1)). It has long been known that a reduction in food consumption, without malnutrition, called dietary/caloric restriction may decrease the risk of number pathologies. Therefore, understanding the mechanisms providing beneficial effects of calorie restriction may provide some clues to wisely use such approaches. Using our proposed classification of oxidative/carbonyl/nitrosative stress (Lushchak, 2014[20], 2015[24]; Garaschuk et al., 2018[11]) helped us to visualize the current experimental data and explain why these data differ from that obtained by other laboratories. One serious conclusion may be drawn from our data: *young* *animals subjected to dietary restriction are exposed to less intensive chronic oxidative/carbonyl stress than their AL counterparts*. If this is true, then one may ask - is it actually good for laboratory animals to be given unlimited access to food? Moreover, these animals are frequently kept in very limited spaces (cages) and with a poor (not stimulating) environment that could also add stress. This leads to new questions. How much food should be provided? At what time(s) should food be delivered? What is the appropriate manner of delivery? Although AL feeding is an easy regimen for the experimentalist to set up, are these animals fully healthy? Clearly, answering these questions could lead to much more work in maintaining animal colonies but the questions cannot anymore be ignored.

## Declaration

### Authorship contribution statement

**Oksana V. Kuzniak: **performance of experiments, formal analysis and data curation.** Oksana M. Sorochynska: **performance of experiments.** Maria M. Bayliak: **data curation and visualization, writing of methods section.** Andrii Ya. Klonovskyi: **performance of experiments.** Yulia V. Vasylyk: **performance of experiments.** Halyna M. Semchyshyn: **data analysis, writing - review and editing,** Kenneth B. Storey: **writing - review and editing.** Olga Garaschuk: **writing - review and editing. **Volodymyr I. Lushchak: **design of the study, writing of original draft, review and editing. 

### Disclosure of interest

The authors report no conflict of interest.

### Ethical statements

All mouse protocols were approved by the Animal Experimental Committee of Vasyl Stefanyk Precarpathian National University (Ukraine) and were conducted in accordance with the European Communities Council Directives of 24 November 1986 (86/609/ECC).

### Acknowledgment

This work was partially supported by the Volkswagen Foundation (VolkswagenStiftung, Germany) under grant #90233 to OG and VIL; the Ministry of Education and Science of Ukraine under grant #0118U003477 to VIL; the grant from National Research Foundation of Ukraine (#2020.02/0118) to MMB; the Natural Sciences and Engineering Research Council of Canada under Discovery grant from #6793 to KBS. The authors also would like to take this opportunity to acknowledge the time and effort devoted by the anonymous reviewer to improve the quality of the manuscript overall and particularly for the advice to add several important issues.

## Supplementary Material

Supplementary data

## Figures and Tables

**Table 1:**
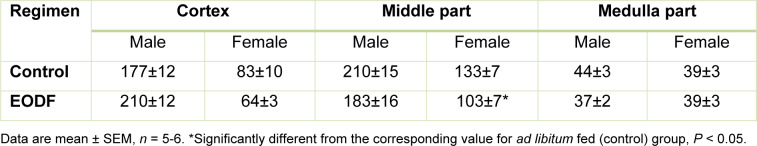
The effect of every-other-day fasting (EODF) regimen on the activities of aconitase (mU/mg protein) in different parts of the brains of young mice

**Table 2:**
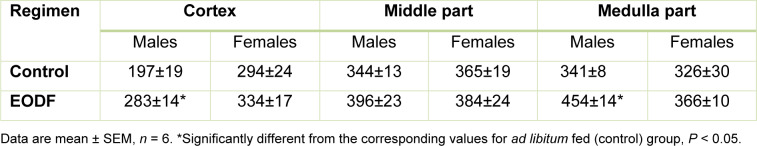
The effect of every-other-day fasting (EODF) regimen on the total non-specific antioxidant capacities (nmol Trolox equivalents/mg protein) in different parts of the brain of young mice
