# Supplementary material for: Feeding to satiation induces mild oxidative/carbonyl stress in the brain of young mice
Source: EXCLI J. 2022 Jan 5;21:77–92. doi: 10.17179/excli2021-4347 (PMC8822308; doi:10.17179/excli2021-4347)
Supplement: Supplementary data [file EXCLI-21-77-s-001.pdf]

EXCLI Journal 2022;21:77-92 – ISSN 1611-2156
Received: September 24, 2021, accepted: December 09, 2021, published: January 05, 2022

Supplementary data to:

Original article:

FEEDING TO SATIATION INDUCES MILD OXIDATIVE/CARBONYL STRESS IN THE BRAIN OF YOUNG MICE

Oksana V. Kuzniak[a], Oksana M. Sorochynska[a], Maria M. Bayliak[a], Andrii Ya. Klonovskyi[a], Yulia V. Vasylyk[a], Halyna M. Semchyshyn[a], Kenneth B. Storey[b], Olga Garaschuk[c], Volodymyr I. Lushchak[a,*]

[a] Department of Biochemistry and Biotechnology, Vasyl Stefanyk Precarpathian National University, 57 Shevchenko Str., Ivano-Frankivsk, 76018, Ukraine
[b] Institute of Biochemistry, Carleton University, 1125 Colonel By Drive, Ottawa, Ontario, K1S 5B6, Canada
[c] Department of Neurophysiology, Institute of Physiology, University of Tübingen, 72074 Tübingen, Germany

* **Corresponding author:** Volodymyr I. Lushchak, Department of Biochemistry and Biotechnology, Vasyl Stefanyk Precarpathian National University, 57 Shevchenko Str., Ivano-Frankivsk, 76018, Ukraine. E-mail: lushchak@pnu.edu.ua

https://dx.doi.org/10.17179/excli2021-4347

This is an Open Access article distributed under the terms of the Creative Commons Attribution License (http://creativecommons.org/licenses/by/4.0/).

EXCLI Journal 2022;21:77-92 – ISSN 1611-2156
Received: September 24, 2021, accepted: December 09, 2021, published: January 05, 2022

Raw data concerning **Table 1:** The effect of every-other-day fasting (EODF) regimen on the activities of aconitase (mU/mg protein) in different parts of the brains of young mice

Supplementary Table 1: Activities of aconitase (mU/mg protein) in different parts of the brains of individual mouse of control and EODF groups

	**Cortext**						**Middle part**						**Medulla part**			
	**Males**		**Females**				**Males**		**Females**				**Males**		**Females**	
**#Mouse**	**Control**	**EODF**	**Control**	**EODF**		**#Mouse**	**Control**	**EODF**	**Control**	**EODF**		**#Mouse**	**Control**	**EODF**	**Control**	**EODF**
**1**	151	197	115	61		**1**		193	149	97		**1**	42.7	29.5	35.8	33.2
**2**	167	222	75	56		**2**	214	177	115	134		**2**	46.0	43.3	40.0	34.6
**3**	157	209	56	57		**3**	249	220	149	103		**3**	30.0	31.4	28.1	40.4
**4**	192	198	91	72		**4**	244	234	128	100		**4**	44.4	35.3	45.9	34.3
**5**	216	173	76	70		**5**	208	146	126	101		**5**	56.8	41.7	45.4	45.8
**6**		261		68		**6**	202	131		80		**6**	44.7	42.9		48.3
**mean**	**177**	**210**	**82.6**	**64.0**		**mean**	**223**	**184**	**133**	**103**		**mean**	**44.1**	**37.4**	**39.0**	**39.4**
**SEM**	**12**	**12**	**9.8**	**2.82**		**SEM**	**9**	**17**	**7**	**7**		**SEM**	**3.5**	**2.5**	**3.3**	**2.6**
**n**	**5**	**6**	**5**	**6**		**n**	**6**	**6**	**5**	**6**		**n**	**6**	**6**	**5**	**6**

EXCLI Journal 2022;21:77-92 – ISSN 1611-2156
Received: September 24, 2021, accepted: December 09, 2021, published: January 05, 2022

Raw data concerning **Table 2:** The effect of every-other-day fasting (EODF) regimen on the total non-specific antioxidant capacities (nmol Trolox equivalents/mg protein) in different parts of the brain of young mice

Supplementary Table 2: Total non-specific antioxidant capacities (nmol Trolox equivalents/mg protein) in different parts of the brains of individual mouse of control and EODF groups

	**Cortext**						**Middle part**						**Medulla part**			
	**Males**		**Females**				**Males**		**Females**				**Males**		**Females**	
**#Mouse**	**Control**	**EODF**	**Control**	**EODF**		**#Mouse**	**Control**	**EODF**	**Control**	**EODF**		**#Mouse**	**Control**	**EODF**	**Control**	**EODF**
**1**	182	250	313	333		**1**	324	348	331	378		**1**	345	452	222	352
**2**	114	266	361	320		**2**	357	336	383	334		**2**	354	445	434	352
**3**	212	250	317	401		**3**	298	361	345	387		**3**	369	462	348	415
**4**	214	327	329	259		**4**	338	420	345	457		**4**	338	460	364	359
**5**	252	320	249	352		**5**	359	480	336	445		**5**	329	506	270	371
**6**	207	285	198	338		**6**	387	432	450	305		**6**	312	397	319	347
**mean**	**197**	**283**	**294**	**334**		**mean**	**344**	**396**	**365**	**384**		**mean**	**341**	**454**	**326**	**366**
**SEM**	**19**	**14**	**24**	**17**		**SEM**	**13**	**23**	**19**	**24**		**SEM**	**8**	**14**	**30**	**10**
**n**	**6**	**6**	**6**	**6**		**n**	**6**	**6**	**6**	**6**		**n**	**6**	**6**	**6**	**6**

EXCLI Journal 2022;21:77-92 – ISSN 1611-2156
Received: September 24, 2021, accepted: December 09, 2021, published: January 05, 2022

Raw data concerning **Figure 2:** The effect of EODF on the levels of markers of oxidative/carbonyl stresses in different parts of the brains of young mice: (**A**) carbonyl groups in proteins (*n* = 4-6), (**B**) lipid peroxides (*n* = 3-6) and (**C**) α-dicarbonyl compounds (*n* = 5-6). Data are mean ± SEM

Supplementary Table 3: Levels of markers of oxidative/carbonyl stresses (carbonyl groups in proteins, lipid peroxides and α-dicarbonyl compounds) in different parts of the brains of individual mouse of control and EODF groups

	**Cortext**						**Middle part**					**Medulla part**			
	**Males**		**Females**				**Males**		**Females**			**Males**		**Females**	
**#Mouse**	**Control**	**EODF**	**Control**	**EODF**		**#Mouse**	**Control**	**EODF**	**Control**	**EODF**	**#Mouse**	**Control**	**EODF**	**Control**	**EODF**
**Carbonyl groups in proteins (nmol/ mg protein)**															
**1**		4.7	6.9	6.4		**1**	1.7	1.8	4.8	4.7	**1**	4.6	3.5	9.1	6.7
**2**	5.1	4.3	6.9	6.4		**2**	2.0	1.7	4.1	4.1	**2**	3.6	4.2	5.4	5.0
**3**	4.8	3.8	8.2	5.2		**3**	1.8	1.5	5.6	3.3	**3**	3.6	4.0	5.4	5.3
**4**	5.8	4.4	8.3	5.9		**4**	2.1	1.7	4.4	3.7	**4**	2.6	2.8	5.3	4.4
**5**	6.6		7.5			**5**	1.8	1.6	5.0		**5**	2.7	2.7	5.1	
**6**	7.6		8.4			**6**		1.6	4.1		**6**		2.0	5.0	
**mean**	**5.98**	**4.3**	**7.7**	**6.0**		**mean**	**1.9**	**1.6**	**4.7**	**3.9**	**mean**	**3.0**	**3.4**	**5.2**	**4.6**
**SEM**	**0.51**	**0.19**	**0.27**	**0.30**		**SEM**	**0.06**	**0.04**	**0.23**	**0.29**	**SEM**	**0.27**	**0.31**	**0.06**	**0.24**
**n**	**5.00**	**4**	**6**	**4**		**n**	**5**	**6**	**6**	**4**	**n**	**5**	**5**	**5**	**5**
**Lipid peroxides (nmol cumene equivalents/gwm)**															
**1**		287				**1**					**1**				332
**2**	353	318	188			**2**		117		440	**2**		287		239
**3**	291	321	308	344		**3**	298	149		225	**3**	353	318	188	256
**4**	393	281	254	371		**4**	208	237	270	125	**4**	291	321	308	301
**5**	305	327	258	187		**5**	89	186	185		**5**	393	281	254	160
**6**		125	173	182		**6**	188		199		**6**	305	327	258	

**mean**	**336**	**277**	**236**	**271**		**mean**	**196**	**172**	**270**	**263**	**mean**	**336**	**125**	**173**	**258**
**SEM**	**23**	**31**	**25**	**50**		**SEM**	**32**	**26**	**26**	**80**	**SEM**	**23**	**277**	**236**	**29**
**n**	**4**	**5**	**5**	**4**		**n**	**4**	**4**	**3**	**3**	**n**	**4**	**31**	**25**	**5**
**Alpha-dicarbonyls (nmol glyoxal equivalents/gwm)**															
**1**	57.0	48.4	70.6	67.8		1	73.7	69.7	59.4	54.1	1	65.1	78.3	60.9	72.9
**2**	58.8	59.2	64.9	72.5		2	56.8	71.7	60.4	47.4	2	71.0	54.4	59.7	73.1
**3**	57.5	50.5	73.3	59.4		3	53.8	67.7	61.0	52.0	3	64.8	59.9	88.4	67.6
**4**	73.8	50.1	68.9	66.4		4	65.9	63.0	85.4	71.6	4	81.1	62.6	65.3	44.5
**5**	66.1	55.2	62.0	59.1		5	72.0	62.0	73.5	66.0	5	76.2	65.5	78.4	40.0
**6**	63.0	66.6	70.8	98.9		6	91.5	61.0	121	61.8	6	92.2	65.1	71.3	43.2
**mean**	**62.7**	**52.7**	**68.4**	**65.0**		**mean**	**64.4**	**65.9**	**67.9**	**58.8**	**mean**	**75.1**	**61.5**	**70.7**	**48.8**
**SEM**	**2.6**	**1.9**	**1.7**	**2.56**		**SEM**	**3.9**	**1.8**	**5.1**	**3.8**	**SEM**	**4.3**	**2.0**	**4.5**	**6.5**
**n**	**6**	**5**	**6**	**5**		**n**	**5**	**6**	**5**	**6**	**n**	**6**	**6**	**6**	**6**

EXCLI Journal 2022;21:77-92 – ISSN 1611-2156
Received: September 24, 2021, accepted: December 09, 2021, published: January 05, 2022

Raw data concerning **Figure 3:** The effect of EODF on the activities of (**A**) superoxide dismutase (*n* = 3-6), (**B**) catalase (*n* = 5-6), (**C**) glutathione peroxidase (*n* = 4-6), (**D**) glutathione-S-transferase (*n* = 4-6) and (**E**) glyoxalase 1 (*n* = 5-6) in different parts of the brain of male and female young mice. Data are mean ± SEM.

Supplementary Table 4: Activities of superoxide dismutase (SOD), catalase, glutathione peroxidase (GPx), glutathione-S-transferase (GST) and glyoxalase 1 (GLO1) in different parts of the brains of individual mouse of control and EODF groups

	**Cortex**						**Middle part**						**Medulla part**			
	**Males**		**Females**				**Males**		**Females**				**Males**		**Females**	
**#Mouse**	**Control**	**EODF**	**Control**	**EODF**		**#Mouse**	**Control**	**EODF**	**Control**	**EODF**		**#Mouse**	**Control**	**EODF**	**Control**	**EODF**
**SOD activity (U/mg protein)**																
**1**	207	214	244	259		**1**		345	256	157		**1**	263	215	118	
**2**	179	160	220	189		**2**	361	386	207	177		**2**	380	200	147	150
**3**	235	235	196	193		**3**	382	245	244	185		**3**	384	189	177	131
**4**	184	141	170	256		**4**	376	220	345	278		**4**			236	76
**5**	177	132	212	142		**5**	215	232	337	337		**5**			242	112
**6**		173				**6**	256		288	369		**6**			211	91
**mean**	**196**	**176**	**208.4**	**207.8**		**mean**	**318**	**286**	**279**	**251**		**mean**	**342**	**201**	**184**	**117**
**SEM**	**11**	**16**	**12**	**22**		**SEM**	**34**	**34**	**22**	**37**		**SEM**	**40**	**8**	**22**	**14**
**n**	**5**	**6**	**5**	**6**		**n**	**5**	**5**	**5**	**6**		**n**	**3**	**3**	**6**	**5**
**Catalase activity (U/mg protein)**																
**1**	4.76	4.60	8.60	6.45		**1**	6.06	6.61	4.92	4.26		**1**	1.71	1.89	1.82	1.25
**2**	4.46	3.01	6.39	6.55		**2**	5.43	6.65	4.35	5.50		**2**	1.35	2.04	1.70	1.31
**3**	5.33	3.88	8.69	6.94		**3**	5.59	4.10	4.82	7.01		**3**	1.71	1.49	1.63	1.31
**4**	5.30	6.73	10.4	8.13		**4**	6.63	4.98	5.43	6.69		**4**	1.99	1.99	1.56	1.15
**5**	5.08	5.95	13.1	8.04		**5**	6.58	4.74	5.34	8.27		**5**	1.26	2.03	2.03	1.27

**6**	4.72	4.78		7.73		**6**		5.56		4.43		**6**	1.65	1.29		1.63
**mean**	**4.94**	**4.83**	**9.44**	**7.31**		**mean**	**6.06**	**5.44**	**4.97**	**6.03**		**mean**	**1.61**	**1.79**	**1.75**	**1.32**
**SEM**	**0.14**	**0.55**	**1.12**	**0.31**		**SEM**	**0.22**	**0.42**	**0.19**	**0.64**		**SEM**	**0.11**	**0.13**	**0.08**	**0.07**
**n**	**6**	**6**	**5**	**6**		**n**	**6**	**6**	**5**	**6**		**n**	**6**	**6**	**5**	**6**
**GPx activity (mU/mg protein)**																
**1**		9.60		37.1		**1**		21.5		16.9		**1**			2.78	8.91
**2**	10.4	24.5	13.4	27.3		**2**	12.2	42.8	19.5	20.9		**2**	12.1	5.98	48.5	54.0
**3**	20.4	10.7	26.5	34.3		**3**	18.8	35.9	22.3	14.0		**3**	9.48	7.99	46.5	46.7
**4**	16.3	6.5	17.6	18.8		**4**	5.7	40.0	9.15	18.7		**4**	13.6	10.9	18.6	49.8
**5**	19.7	18.8	16.4	24.4		**5**	10.2	19.8	8.61	17.4		**5**	11.0	4.01	46.0	35.1
**6**	14.3		22.5	17.8		**6**		29.8	22.6			**6**	13.8	14.0		33.2
**mean**	**16.2**	**14.0**	**19.3**	**26.6**		**mean**	**11.7**	**31.6**	**16.4**	**17.6**		**mean**	**12.0**	**8.6**	**39.9**	**43.8**
**SEM**	**1.8**	**3.3**	**2.3**	**3.2**		**SEM**	**2.7**	**3.9**	**3.1**	**1.2**		**SEM**	**0.8**	**1.8**	**6.4**	**4.1**
**n**	**5**	**5**	**5**	**6**		**n**	**4**	**6**	**5**	**5**		**n**	**5**	**5**	**4**	**5**
**GST activity (mU/mg protein)**																
**1**		80	153			**1**	195	192	225	228		**1**	207		182	249
**2**		111.0	224	214		**2**	176	138	222	174		**2**	243	79.7	202	269
**3**	221	132	229	225		**3**	193	261	267	219		**3**	239	174	238	286
**4**	254	299	81.2	115		**4**	128	191	189	237		**4**		32.9	187	217
**5**	234	292	103	149		**5**	147	231	212	189		**5**	175	184	204	238
**6**	252	222	138	112		**6**	127		221			**6**	208			234
**mean**	**240**	**211**	**155**	**163**		**mean**	**161**	**203**	**223**	**209**		**mean**	**214**	**118**	**203**	**249**
**SEM**	**8**	**39**	**25**	**24**		**SEM**	**13**	**21**	**10**	**12**		**SEM**	**11**	**30**	**9**	**10**
**n**	**4**	**5**	**6**	**5**		**n**	**6**	**5**	**6**	**5**		**n**	**5**	**4**	**5**	**6**

EXCLI Journal 2022;21:77-92 – ISSN 1611-2156
Received: September 24, 2021, accepted: December 09, 2021, published: January 05, 2022

**GLO1 activity (mU/mg protein)**															
**1**	2.98	3.30	3.62	3.40		**1**	3.94	4.61	3.96	4.78		**1**	2.84	5.74		4.51
**2**	3.56	3.72	3.78	4.08		**2**	4.02	3.93	3.89	3.77		**2**	3.98	3.95	4.48	5.25
**3**	3.92	3.27	3.87	4.03		**3**	3.55	3.92	4.04	3.78		**3**	4.91	3.54	5.07	5.62
**4**	2.76	3.71	2.81	2.99		**4**	4.70	4.06	3.96	4.32		**4**	3.72	5.26	3.92	4.37
**5**	3.60	3.98	2.83	3.14		**5**	4.19	5.10	4.69	4.63		**5**	4.59	5.43	3.99	4.04
**6**	2.90		3.55	3.10		**6**	4.84	4.56		3.74		**6**	4.76	5.68	4.18	5.30
**mean**	**3.29**	**3.60**	**3.41**	**3.46**		**mean**	**4.21**	**4.36**	**4.11**	**4.17**		**mean**	**4.13**	**4.93**	**4.33**	**4.85**
**SEM**	**0.19**	**0.14**	**0.19**	**0.20**		**SEM**	**0.20**	**0.19**	**0.15**	**0.19**		**SEM**	**0.32**	**0.39**	**0.21**	**0.26**
**n**	**6**	**5**	**6**	**6**		**n**	**6**	**6**	**5**	**6**		**n**	**6**	**6**	**5**	**6**

Raw data concerning **Figure 4:** The effect of EODF on the activities of the enzymes responsible for maintaining glutathione redox status: (**A**) glutathione reductase (*n* = 5-6), (**B**) glucose-6-phosphate dehydrogenase (*n* = 4-6) and (**C**) NADP-dependent isocitrate dehydrogenase (*n* = 4-6) in different parts of the brain of young mice. Data are mean ± SEM.

Supplementary Table 5: Activities of glutathione reductase (GR), glucose-6-phosphate dehydrogenase (G6PDH) and NADP-dependent isocitrate dehydrogenase (IDH) in different parts of the brains of individual mouse of control and EODF groups

	**Males**		**Females**				**Males**		**Females**				**Males**		**Females**	
**#Mouse**	**Control**	**EODF**	**Control**	**EODF**		**#Mouse**	**Control**	**EODF**	**Control**	**EODF**		**#Mouse**	**Control**	**EODF**	**Control**	**EODF**
**GR activity (mU/mg protein)**																
**1**	5.52	5.14	9.63	10.7		**1**		10.6	12.1	10.0		**1**		15.7	13.8	19.0
**2**	5.97	7.33	9.93	9.93		**2**	8.22	9.31	12.8			**2**	17.5	12.8	18.2	20.7
**3**	7.94	6.43	10.1	10.6		**3**	10.7	10.4	11.7	11.2		**3**	14.6	17.9	21.4	13.5
**4**	9.83	15.7	7.21	7.69		**4**	11.9	10.8	13.4	13.7		**4**	22.7	11.5	20.9	15.6
**5**	12.3	13.5	8.26	6.90		**5**	11.7	9.05	10.8	10.9		**5**	21.9	13.2	16.9	11.6
**6**		12.5	9.81	8.07		**6**	11.7			8.49		**6**	18.5	9.83	16.1	12.5
**mean**	**8.31**	**10.10**	**9.16**	**8.98**		**mean**	**10.8**	**10.0**	**12.2**	**10.6**		**mean**	**19.0**	**13.5**	**17.9**	**15.5**
**SEM**	**1.26**	**1.77**	**0.47**	**0.67**		**SEM**	**0.7**	**0.3**	**0.4**	**0.8**		**SEM**	**1.5**	**1.2**	**1.2**	**1.5**
**n**	**5**	**6**	**6**	**6**		**n**	**5**	**5**	**5**	**5**		**n**	**5**	**6**	**6**	**6**
**G6PDH activity (mU/mg protein)**																
**1**	6.11	7.71		18.1		**1**	27.7	22.6		20.4		**1**	20.2	14.6	39.5	31.7
**2**	9.25	11.0	15.9	14.0		**2**	25.7	22.6	28.1	21.5		**2**	19.1	16.9	32.1	40.8
**3**	12.8	10.7	12.4	12.2		**3**	24.7	25.0	23.8	15.2		**3**	17.2	21.2	32.5	42.7
**4**	17.0	19.1	18.0	28.0		**4**	25.5	25.4	28.6	18.9		**4**	23.8	21.3	33.4	34.0
**5**	14.0	12.7	23.0	29.9		**5**	23.8	26.3	30.8	15.8		**5**	18.8	13.1		33.3
**6**	15.5	14.9	21.7	34.7		**6**	27.6		32.7			**6**	20.4	24.5		29.4

EXCLI Journal 2022;21:77-92 – ISSN 1611-2156
Received: September 24, 2021, accepted: December 09, 2021, published: January 05, 2022

**mean**	**12.4**	**12.7**	**18.2**	**22.8**		**mean**	**25.8**	**24.4**	**28.8**	**18.4**		**mean**	**19.9**	**18.6**	**34.4**	**35.3**
**SEM**	**1.7**	**1.6**	**1.9**	**3.8**		**SEM**	**0.6**	**0.8**	**1.5**	**1.2**		**SEM**	**0.9**	**1.8**	**1.7**	**2.1**
**n**	**6**	**6**	**5**	**6**		**n**	**6**	**5**	**5**	**5**		**n**	**6**	**6**	**4**	**6**
**IDH activity (mU/mg protein)**																
**1**		24.6	20.4			**1**	14.6	21.2	32.0	18.3		**1**	11.6	17.0		10.8
**2**	19.7	19.1	19.2	22.2		**2**	14.9	25.8	30.5	30.5		**2**	12.6	8.29	9.37	7.46
**3**	21.7	27.6	19.1	21.0		**3**	19.1	20.3	20.9	23.2		**3**	19.2	15.1	9.13	8.62
**4**	21.3	26.5	18.2	12.4		**4**	22.3	21.8	23.0	33.8		**4**	22.2	7.80	11.9	10.8
**5**	20.1	27.4	13.3	17.3		**5**	19.5		20.4	17.7		**5**	15.8	12.0	11.4	7.44
**6**	22.8	35.6	18.4	13.6		**6**	15.0					**6**	15.2			6.36
**mean**	**21.1**	**26.8**	**18.1**	**17.3**		**mean**	**17.6**	**22.3**	**25.4**	**24.7**		**mean**	**16.1**	**12.0**	**10.5**	**8.6**
**SEM**	**0.5**	**2.2**	**1.0**	**1.9**		**SEM**	**1.3**	**1.2**	**2.5**	**3.2**		**SEM**	**1.6**	**1.8**	**0.7**	**0.8**
**n**	**5**	**6**	**6**	**5**		**n**	**6**	**4**	**5**	**5**		**n**	**6**	**5**	**4**	**6**
